
JOURNAL INFORMATION
==============================
NLM Title Abbreviation: Med Chir Rev
NLM Journal ID: 0034617

The Medico-Chirurgical Review

Publisher: Biomedical Journal Digitization Project

ARTICLE INFORMATION
==============================
PMCID: PMC4966251
PMID: 29917623
Article version: 1
Subjects: Articles

Hospital Practice

Print publication date: 1828 Oct 1
Volume: 9
Issue: 18
First page: 444
Last page: 471
1828 Jul
License: This work is free of known copyright restrictions. For more information, please see PMC Back Issue Digitization.
License URL: https://creativecommons.org/publicdomain/mark/1.0/

==============================

occasioned. Serous effusions were the natural results. There is reason to infer, that the biliary secretion was suspended. The ammoniacal state of the urine is the usual consequence of the spinal accident; and inflammation of the bladder is often the cause of death. We may here remark, that in cases of paraplegia from spinal injury, the introduction of the catheter is frequently productive of great mischief. The parts are insensible; and the inexperienced house-surgeon (when the patient is in hospital) works away, and thinks he is doing no harm while the patient does not complain. The effects appear, sooner or later, in the shape of inflammation or sloughing of the urinary passages. Mr. Bell has noticed this, among many other excellent observations of that talented surgeon.

"Was the discharge from the bowels on the fourth day the result of the action of the tartar emetic, or was it the consequence of the injury of the spinal marrow? When a bladder has been paralyzed from injury of its nerves, retention of urine is the immediate result, but sooner or later this is followed by incontinence, as was the case in the present instance. This phenomenon is to be explained by a law to which the action of nerves appears to be subject, viz. one degree of pressure or irritation will produce spasm or convulsion, and a greater degree of the same will cause loss of power, or paralysis. This law will be found to govern the muscular system of both voluntary and involuntary action, and consequently the sphincters." 219.

It need not excite surprise, in this period of surgical science, that the patient should have lived so long after complete division of the spinal marrow. Independent of Magendie's celebrated case, where a considerable portion of spinal marrow was found obliterated, and yet where sensation and motion in the lower extremeties remained, we believe that Mr. Bell met with a case of complete division of the "spinal brain," and where the patient lived nearly a year;—and died, at last, of some eruptive disease—and not at all of the spinal injury. It is more than probable, that had Mr. Bell's patient not died of another disease, a considerable degree of sensation and motion would have returned—by a kind of collateral circulation of the nervous energy, in the same way that the circulation of the blood is carried on by circuitous routes, when the regular channel is blocked up.

---

### HOSPITAL PRACTICE.

### ST. GEORGE'S HOSPITAL.

### 13. INJURIES OF THE HEAD.

Sir Astley Cooper was the first to draw a distinction between simple and compound fracture of the skull, a distinction on which is hinged a very material difference of treatment. It has been argued, that this distinction is imaginary, and that there is no analogy whatever between fractures of the cranium and fractures of the long bones. The objection is more specious than solid. If there be a compound fracture of the femur, will any one be so bold as to affirm, that the danger which ensues is merely dependent on the injury of the *bone?* Does the patient die of caries of the *bone*, of necrosis, or exfoliation, or rather, when he sinks, is it not by reason of the mischief to the soft parts, the blood-vessels, the nerves, the muscles, or the tendons? Are the wasting suppurations which so frequently destroy him, in the bone or the parts around? Most undoubtedly the latter; though, of course, they are aggravated by the injured condition of the former.

In fractures of the cranium, the mischief is not in the parts without, but those within—the meninges, or the brain itself. The membranes of the brain are serous membranes, and extremely prone to inflammation, whilst they are as carefully excluded by nature from the contact of the air, as the parts around the femur, the tibia, or any bone whatever. The same thing holds with the brain itself, and yet there are some who deny that it can make the slightest difference, in fractures of the cranium, whether the membranes or the brain are exposed to the atmospheric influence, or not.

We contend, then, that there is a very close analogy between fracture of bones in general, and fracture of the cranium, the danger in both materially depending upon the injury inflicted on, or the inflammation aroused in, the textures in the neighbourhood. In fracture of the long bones, the mischief is done to the parts *around*, in fracture of the cranium, to those *within*, and that is all the difference. There are, however, certain bones, or sets of bones, in which even the above inconsiderable distinction, we can scarcely call it difference, does not exist. We allude to the ribs and the bones of the pelvis; the danger of a broken rib, as every body knows, arising from inflammation of the pleura, which danger is of course increased by the fracture being compound. We might continue to argue on the subject until we wearied ourselves and our readers, but we hope we have said enough to shew, that the distinction which was drawn by Sir Astley Cooper, between simple and compound fractures of the skull is neither void of analogy, nor unfounded in principle. We shall now detail a case of compound fracture of the skull, in which the trephine was had recourse to, and the patient never had a "symptom."

*Case.* William Middleton, æt. 40, was admitted, May 30th, 1828, at 1 a. m. under the care of Mr. Brodie.

He was crossing a field between 8 and 9 o'clock the previous evening, when he witnessed a row between some Irishmen and a blacksmith. He and his companions began to mediate between the parties, when the Irishmen turned upon the peace-maker, and felled him to the ground by a blow with an axe upon the head. He was stunned for upwards of an hour, and, on recovering his senses, he found that both friends and foes had very valiantly made off, and left him master of the *field*, or, at least, lying on it! The wound was bleeding furiously, and after a little while he was carried to a public-house and seen by a practitioner. At midnight, he was brought to St. George's Hospital, and before his arrival the hæmorrhage had ceased.

When admitted, he was perfectly sensible, though weak—no stertor—no paralysis—pupils natural. Three scalp-wounds were discovered, one upon the right side near the ear, another on the opposite side, a little above the temporal ridge of the parietal bone, and the largest of all at the vertex, a little to the right of, and crossing obliquely over the sagittal suture. It was a clean cut, like what would be inflicted by an axe, the bone was depressed and driven apparently into the longitudinal sinus, and the finger could be passed in for half an inch, but touched neither brain nor dura mater. The other wounds were superficial—the lower jaw was broken.

He was ordered a purgative of calomel and jalap, and continued without any untoward symptoms throughout the day. At 10 p. m. Mr. Brodie saw him for the first time, and although no symptoms had appeared, applied the trephine and removed the pieces of depressed bone. The inner table was more extensively broken than the outer, causing some little difficulty in its elevation. The edges of the wound were brought together with a suture, and lightly dressed.

He complained of a little pain in the head after the operation, but it soon went off, and he passed a quiet night. In the afternoon of the succeeding day, he had some shivering and nausea, with a quick pulse, and whitish tongue. These symptoms subsided in the evening, but considerable purging came on during the night. He was bled at 6 p. m. to twelve or fourteen ounces, and a spirit lotion put upon the head. The purging in a day or two subsided of itself, and indeed on the 3rd of June it was necessary to order calomel and senna draught to procure a stool. No bad symptoms whatever supervened, the wound in the scalp granulated kindly, and the patient will shortly be dismissed the Hospital.

In this case there cannot be a doubt that the longitudinal sinus was extensively opened into, notwithstanding which, the bleeding was comparatively trifling. The absence of symptoms from the first would lead one to imagine, that the depressed bone had been forced into the sinus, but had not injured the dura mater, and membranes immediately covering the brain. Whether inflammation of the membranes would have come on if those bones had not been raised at all, can of course, be only matter of conjecture, but the probabilities are surely more in favour of its occurrence, when spiculæ of bone are in contact with and irritate the dura mater, than when they are elevated and removed.

In an interesting clinical lecture delivered on the case by Mr. Brodie, he remarked, that it not unfrequently happens, in fractures with depression, that suppuration takes place between the bone and the dura mater, and if the collected pus has not a ready exit, inflammation ensues in the deeper-seated membranes, opposite the point where the matter is pent up. Mr. Brodie agreed, in the main, with Sir Astley Cooper, on the propriety of a distinction between simple and compound fractures of the cranium, and observed that he had many cases, some of which he had seen himself, and others he had collected from various sources, which went to establish the position of Sir Astley. There is, in fact, said Mr. B. a considerably greater danger of suppuration between the bone and the dura mater when the scalp is wounded, than when it is entire. On this account, as well as to give a readier egress to the pus, if it should collect, Mr. Brodie had recourse to the trephine. Mr. Brodie gave an additional reason for the operation, viz. that when the broken bone is allowed to remain depressed, the patient may recover in the first instance, but be affected at last with a series of anomalous symptoms, as convulsions, paralysis, or numbness, when he returns to his usual avocations and habits of life. If the scalp be wounded and bone depressed, but a considerable interval remain between the broken fragments, Mr. Brodie does not consider the operation so decidedly called for, as the pus, if it forms upon the dura mater, is already provided with the means of escape. In confirmation of this remark, Mr. B. alluded to the case of a boy, who was admitted with symptoms very similar to those in the present instance. The trephine was not applied, but in the course of several days feverishness and unpleasant symptoms supervened. The wound had partially united, preventing the matter from escaping freely; the scalp was divided, the collected pus let out, and the patient did extremely well.

In the above case, the bone was fractured and depressed, but in the one we are going to relate it was merely denuded, though there were reasons for supposing, at the time, that more or less extravasation had taken place within the cranium.

*Case* 2. John Antcliff, ætatis 37, was brought into St. George's Hospital, on the 2d of June, at 3 P.M. having fallen from a hay-loft, twenty feet in height.

He was perfectly insensible, muttering some indistinct exclamation, when loudly holloa'd to, but evidently incapable of understanding what was said. The pulse was soft and slow, the surface cool, the whole frame relaxed, the pupils fixedly dilated. Over the left ear, there was a considerable scalp-wound, exposing a portion of the parietal bone, and there were several bruises on other parts. There was reason to suspect that the patient was in liquor. In the evening some re-action had appeared, and on the 3d, though drowsy he was sensible when roused; the pulse was 86, labouring and hard.—*Haustus sennæ.—V. S. ad* ℥ xij.—*Saline with salts and antimonial wine every four hours.*

The bleeding was repeated in the evening, and again upon the 4th, when he appeared to be in a very precarious condition. He was lethargic, and incapable of being properly aroused; scarcely comprehended any question put to him, or muttered an incoherent answer, and was extremely restless, constantly arising out of bed and tumbling the bed-clothes. The bowels were purged, the pupils dilated, the wound puffy, and there was œdema of the scalp around. On the 5th the symptoms were in some degree relieved, but he was, at times, delirious, and had passed an unquiet night.—*Cataplasma lini capiti raso.*

*6th.* Worse than yesterday, and was quite delirious in the night. He sits in a moping moody state, scarcely answering a question, and that in a low desponding tone; pupils dilated; pulse small and low; pain in the head, especially in the situation of the wound, which is very sloughy.—*V.S. ad* ℥ x.

He had a comfortable night and was better on the 7th. On the 8th, the pupils acted freely, the head-ache gradually subsided, and on the 14th he left the hospital, though against the wish of Mr. Brodie, who thought it extremely probable that he would soon return, with a re-appearance of the symptoms. This prognosis was prophetic, for on the 19th, the man was re-admitted, with head-ache and giddiness; countenance dull and sodden; pulse full and hard; tongue brown. He was purged and bled to ten or twelve ounces, and the head-ache and giddiness disappeared in a day or two. At present he is doing well.

Mr. Brodie was inclined to believe, from the obstinacy and severity of the symptoms, that a slight extravasation had occurred within the head. At one time, indeed, Mr. B. thought it not improbable that matter might form on the surface of the dura mater, opposite the point where the bone was denuded, and require the application of the trephine. Fortunately this was not the case, or the chances of recovery would have been but slight, as the following account will prove.

*Case* 3. A man of good constitution, and about thirty-six years of age, received a blow upon the head, from the stool on which he was sitting being overturned. A scalp-wound was the consequence, by which the cranium was denuded at its upper part, but not a symptom either of concussion or compression in the first instance supervened. The inflammation in the scalp was treated by the proper remedies, but at the expiration of a week or ten days, the patient was seized with shivering, vomiting, and a fever which was considered intermittent, and treated, we need scarcely say without success, by the sulphate of quinine. The symptoms went on, and in the course of a few days he was suddenly attacked with hemiplegia of the left side, and carried to the Hôtel Dieu.

The paralysis, the stupor without total loss of sense, the history of the accident, and the progress of the symptoms, led M. Sanson and M. Dupuytren to conclude that pus was effused either in the brain, or on the surface of the dura mater. There was neither fracture nor depression of the cranium, and had there been extravasation, there would have been some symptoms of its presence from the first. Believing, then, that the exposure and denudation of the bone had caused inflammation and separation of the dura mater, with effusion of pus either above it or below it, the trephine was applied over the denuded part by M. Sanson, with the entire concurrence of his colleague.

Before the whole circle of bone embraced in the trephine was got away, a drop or two of pus was seen oozing up beside its margin, and at length, when the bone was removed, the dura mater was perceived to be covered with a purulent exudation, which adhered very firmly to its surface. The dura mater itself was glistening, and not at all thrown upwards, or raised above its natural level, as it would be if pus were collected in any considerable quantity beneath it. This being the case, no incision was made into the membrane, but the operation was concluded, and the patient sent to bed. After the man was removed from the theatre, M. Dupuytren remarked that the chances of success were extremely slight, as the dura mater, in all probability, was detached from the cranium to a considerable extent, whilst the firmness of the purulent effusion on its surface would effectually prevent its escape by the opening made by the trepan. The tension, without any elevation, of the dura mater prevented the idea of a purulent depôt being placed beneath, but if relief was not obtained, the surgeon might make an incision into the membrane in the evening, without adding materially to the danger of the patient. In M. Dupuytren's opinion, the contact of the external air with the membranes of the brain must exercise a deleterious influence, increase the inflammation, and constitute, in fact, the most frequent cause of the want of success which attends the operation of the trephine.

The paralysis and delirium, as well as the other symptoms, continued to increase, but no incision was made into the dura mater, as the pulsations of the brain were distinguished immediately beneath it, and nothing appeared to indicate the existence of pus.

On the 20th of April the patient died, when dissection discovered a layer of pus *on the surface of the brain*, commencing at least an inch and a half from the spot where the trephine had been applied, and extending as far as the commissura magna, near the border of which the brain was depressed by a little abscess. The arachnoid was injected and inflamed; there was no fracture whatever of the bone, and the purulent effusion on the dura mater had entirely disappeared — *Clinique*.

The above is calculated to illustrate the generally hopeless nature of the operation of trephining, when performed for what may be designated the "secondary symptoms" of injuries of the head. When our object is to elevate a depressed and broken bone, we remove in a great degree the cause of *future* mischief, and operate *before* the fatal inflammation has come on. When, however, we employ the trephine, as we would a lancet in the softer textures, merely for the purpose of *puncturing* the bone and giving issue to a collection of pus upon the dura mater, we are removing not the cause but the consequence of inflammamation which has extended already too widely and deeply over the membranes or the brain itself.

Some of the symptoms which occasionally follow an injury of the head or disease within the cranium are extremely curious. Thus, all must recollect the case of the labourer, who, after a blow upon the head, was admitted into St. Thomas's or Guy's, and found by Sir Astley Cooper jabbering a language that none could understand, until a Welsh woman pronounced it to be admirable Welsh! The patient himself being a Cambrian, the injury of the brain had driven the English entirely "out of his *head*," and left him only the memory of his native tongue. A somewhat similar case has lately occurred to M. Dupuytren at the Hôtel Dieu.

*Case* 4. A young man, about twenty years of age, a miner by profession, fell from a height of a dozen feet, alighting on his heels, but receiving such a shock that he was insensible for half an hour, and unable to articulate distinctly. At the expiration of a couple of years he was brought to the Hôtel Dieu, being supposed to labour under hemiplegia.

The patient was taken into the operating theatre, and examined before the students and gentlemen assembled. He was emaciated, pale, of the middle height, rather embarrassed in his manner, but not having the least appearance of idiotcy or feebleness of intellect.

What is your profession? said the Baron.

*Mine*—answered he with considerable difficulty, and it was only after repeated efforts that he was able to articulate *mineur*.

What age are you?

*Ving—t—t—d—eux—ans.*

Your name?

*Jacques Col—in—Col—as—Col—ard.*

Have you any brothers and sisters? *Oui.* How many brothers? *Un.* How many sisters? *Trois.* What is your father? *Peintre.* What profession is the husband of your eldest sister? *V V Ver—*, vitrier, (a glazier?) asked M. Dupuytren. He shook his head. Does he make bottles? Sign in the negative; and *V V Ver—* was all he could pronounce.*

Do you understand what I am asking you? said the surgeon. *Oui.* Strike the table—he struck it; lift up your foot; put it back upon the ground; turn your head to the right side, to the left; walk forwards; return; put on your cap, &c. all was obeyed with the most military precision. The muscular motions of the tongue were free enough, and it was evident that nothing like paralysis or hemiplegia existed.

On being ordered to repeat *sa, se, si, so, su*, he was unable to articulate the two latter, and merely pronounced the *o* and *u*. Instead of *mon père*, he could only answer *pé—*; for *ma mère*, he answered, *mé—*. The examination was completed, and the patient walking off without a salutation, when M Dupuytren called him back, and told him to doff his cap. He did as he was bid. Bid the gentleman *adieu; ad—d—eu*, said he, and walked away.

On a subsequent day, experiments were made to ascertain whether the tongue, as the organ of *taste*, continued perfect in its functions. Salt, sugar, and pepper were the substances employed; the first of which he recognized, the second he confounded with the first, and the pepper he called rum. On giving him some water he knew and drank it without the slightest difficulty.

---

* This affection of the tongue appears to be an aggravated species of stammering. In a relation of our own, the above disagreeable infirmity was produced by a fall upon the head when a child, and has continued with the greatest obstinacy ever since.

The patient, as was observed before, evinced no signs, whatever, of feebleness of intellect, and the muscular motions of the tongue were free and unembarrassed. M. Dupuytren was induced to consider the affection as resembling what occasionally occurs after apoplexy, or chronic affections of the brain, where the patient suffers loss of the memory of things, or particular words, as proper names, substantives, or adjectives. In some individuals, the power of judging and comparing objects is destroyed, and the reporter himself was acquainted with an old lady, a hemiplegiac, who preserved the use of her intellectual faculties, but could only answer to whatever question she was asked: *Saint Antoine, Saint Antoine!* M. Dupuytren compared the present case to the above, and considered the affection of the tongue, (both as an organ of articulation and of taste,) as rather depending on a general affection of the brain, than on a local lesion of the two sets of nerves which endow the organ with the properties of taste and motion.—*Clinique.*

---

### ST. BARTHOLOMEW'S HOSPITAL.

### 14. Injuries of the Spine.

A strong muscular man fell from a height of 30 feet upon a tomb-stone, with his neck bent under him, and immediately afterwards was admitted into St. Bartholomew's Hospital, with all the symptoms of fracture of the cervical vertebræ, and some of those of concussion of the brain. Warm brandy and water was given him, and hot bottles placed upon the feet. At six next morning, he took a dose of calomel and jalap, and his water was drawn off, and at 10, the pulse being quick and hard, he was bled to ℥xij. When Mr. Earle first saw the patient, at 12 o'clock, the breathing was performed by the diaphragm, but he "could use his arms and hands with some power." Rest was enjoined, but at 9 p. m. the man made some sudden effort, and instantly expired.

On dissection, the spinous processes and arch of the 4th and 5th cervical vertebræ were fractured and slightly displaced, the body of the 4th much comminuted, and projecting forwards, whilst the ligaments of the right articular process had given way so as to admit of subluxation.

This case has formed the ground-work of a clinical lecture, by Mr. Earle, in which he condemns the operation of trephining the spine for fracture of the vertebræ, but as he merely adduces the arguments which were long ago brought forward by Charles Bell, we shall pass them by. Mr. Earle would seem to be somewhat of an optimist in fractures of the spine, or rather his prognostics are of a much more favourable cast than are entertained by the generality of his brethren. For our own parts, we should be led, from the cases we have witnessed, to entertain the very feeblest hopes, where fracture had decidedly occurred. If merely a spinous process be broken off, and the symptoms depend on the concussion which the marrow has received, the patient may do well, but when the fracture has extended to the bony arch or body of the vertebra, as almost always happens, the chances seem to us to be a hundred, aye! a thousand to one against recovery. Mr. Earle, we fear, is scarcely sceptical enough in admitting facts and deducing inferences. Thus, he cites the present case, where the spinous processes and arch of two cervical vertebræ were broken and displaced, and the body of one not only broken, but actually comminuted, as one in which "it was *far from improbable* that the gradual amendment in the motion of the arms would have proceeded to perfect restoration, but for the untoward movement which arrested life."

We remember reading in the Johnsoniana (we allude to the *Samuel* Johnsoniana) a reply of the celebrated bear and lexicographer to Boswell, on the latter's declaring, that whether the Doctor believed some disputed point or not, *he* was full of faith. "Bah! Sir!" was the courteous answer; "what will fill a pint pot, won't fill a quart!" In the present instance we must play the quart pot! The facts that fill Mr. Earle are not enough for us.

---

### HOPITAL DE LA CHARITE.

### 15. Dissection Wounds.

A Scotch medical student, having wounded the thumb of the right hand with a rib, the part inflamed and swelled, the swelling extended to the hand and fore-arm, and deeply-seated abscesses ensued. Incisions through the fascia were made into the hand, the fore-arm and lower part of the arm, excessive suppurations followed, counter-openings were required in various directions, and at length the wounds were so far reduced in size, that the patient was able to leave the Hospital for the sake of country air.

A young man, a joiner, about thirty years of age, was sawing a piece of wood, when the saw escaped from its place, and slightly wounded the left thumb. Severe inflammation attacked the hand and fore-arm, and a few days afterwards, the patient was admitted, the wound on the thumb being nearly healed, but the hand and fore-arm considerably swollen. Deep-seated fluctuation being felt over the ball of the thumb, an incision was made into the part, and some purulent matter discharged. In a day or two, an obscure fluctuation was discovered on the anterior aspect of the fore-arm, an incision deeply made, and issue given to a considerable quantity of pus. The abscess was seated in the cellular tissue beneath the fascia, and extended as deeply as the bones and interosseous ligament. *Strict regimen—poultices of linseed meal.*

On the following days, the tumefaction of the hand and fore-arm had not diminished; the tendon of the flexor longus pollicis was exposed, and came away in sloughs; the wound of the fore-arm itself had a sloughy aspect, and furnished a foul discharge; and lastly, the radio-carpal joint became affected, and its cartilages appeared to be destroyed, or destroying by ulceration. There were, besides, much general fever, frequent shiverings, a little cough, slight pain on the right side of the thorax, and right shoulder, and a dull sound on percussion of that side of the chest. M. Roux was afraid of the existence of pleuritis, but nevertheless determined on amputation of the limb, which was performed upon the 12th of November last.

The steps we need not describe, but we may mention that it was at first attempted to remove the fore-arm at the elbow joint, but afterwards the humerus was sawn across above its condyles, which rendered the operation extremely tedious. On examining the limb, it was found that the muscles on the anterior surface of the fore-arm were completely dissected by the suppuration which had taken place. The abscess reached as high as the upper third of the fore-arm, and its bottom was seen to be formed by the interosseous ligament, the radius, which was bare and scabrous, and the flexor pollicis, which was entirely detached. The tendon of the above muscle was also exposed, and sloughing in the hand, and there was a collection of purulent matter beneath the aponeurosis. The flexor tendons under the annular ligament were bathed in pus, and the radio-carpal joint was filled with it, whilst the articulating surfaces of the radius and carpal bones were denuded of their cartilages in several places.

On the morning of the 13th, the patient was extremely low, the breathing was oppressed, and in the night of the 14th he expired.

*Dissection.* The surface exhibited a bilious tinge. On opening the thorax, there were found to be some old adhesions between the pleuræ of both sides, and behind these were evident traces of pleuro-pneumony. There were also some recently-formed false membranes, a considerable effusion of sero-purulent and sanguineous fluid, whilst the posterior border of both lungs was in a state of hepatization and infiltrated with pus in several points.—*Clinique.*

This was a melancholy case, and had the surgeon been aware of the condition of the lungs, he would never have exposed his patient to the pain and misery of a protracted and useless operation. We cannot, indeed, imagine, that there were any reasonable grounds for resorting to the knife, independent of the disease which existed in the chest. An injury had been received, and given rise to diffused inflammation in the deep-seated cellular tissue, which inflammation would in all probability attack the stump. The secondary operation in compound fractures is widely different, for in that case all active inflammation has subsided, the part is in a great measure reduced to the condition of a suppurating, scrofulous joint, and the amputation is performed to relieve the constitution of a local irritation, and a wasting discharge.

The symptoms of pleuritis were so evident, that M. Roux should have gone further than *suspecting* its existence, he should have *pronounced* it. There is doubtless a disposition to the formation of tubercular deposits in the liver after serious injuries or operations, but for one case in which the liver is affected, there are three or four in which the respiratory apparatus is attacked. It is astonishing, indeed, how prevalent is inflammation and abscess in the lungs, as a consequence of surgical operations, and how insidiously they carry off the patient. We have lately seen two cases, in which they proved the immediate cause of death though during the life-time of the individual, they were not at all suspected. This should put the practitioner on his guard, and induce him to attend with the greatest anxiety to the condition of the chest. There is often little more than a slight dyspnœa; scarcely ever are the symptoms of pleuritis marked. Depletion should be employed with a liberal hand, and further than a person not aware of the insidiousness of the disease, would think was indicated by the symptoms, or even warranted.

### 2. ABSCESS OF THE BREAST.

Phlegmonous abscess of the mamma (of course we are not alluding to what is called milk-abscess) is not extremely common, but still it is more so than is frequently imagined, and has been mistaken for scirrhus of the breast. Much variety of opinion has existed on the preferable mode of treating abscesses in situations which are commonly exposed, as the breast or neck, especially in females. Some have recommended a small incision, some a large incision, and others none at all; but for our own parts, we think, like the camelion in the fable, that all are right, and all are wrong. If the practitioner is consulted early, he will frequently succeed in preventing any considerable accumulation of matter, by placing the patient on a depleting regimen, administering saline purgatives, and keeping the mamma moderately warm. If matter has formed to the extent of rendering resolution unlikely to take place, we should certainly prefer a moderate incision, made transversely, as soon as any redness, or discolouration of the skin appears. Of course the incision should be made as low, or as much towards the axilla as is compatible with the evacuation of the pus. A fomentation should be used, as colder applications can seldom be endured. If the abscess should have gone to a great extent, burrowing in the substance of the gland or cellular membrane which surrounds it, the incisions must be freely made, as the following case will shew.

A woman, thirty years of age, received a violent blow upon the left breast, which soon became extremely swollen. At the end of a month, she was received into La Charité, the breast being enormously swollen, hard, and painful; the integument red and glistening, and fluctuation perceptible, though deeply-seated. Two large incisions were had recourse to, and gave issue to a quantity of healthy pus, evidently from the centre of the gland itself. The opening of the abscess gave relief, but at the expiration of several days, another collection of purulent matter was discovered, which appeared to have no connexion with the former; a free incision was made in this direction, the lancet passing into the midst of the cellular tissue encircling the gland, and in the course of a few days more, both of the abscesses being discovered by the probe to communicate deeply with each other, the communication was enlarged, and kept so by means of lint. The suppuration soon diminished, and the breast regained its natural volume.

The rapidity of the cure in this instance, is very fairly to be attributed to the free incisions which were had recourse to.

---

### HOPITAL DE LA GARDE ROYALE.

### 16. TREATMENT OF CALLOUS ULCERS BY SHEET-LEAD.

Ulcers, from time immemorial, have been considered the opprobrium of our art, and the surgeon or doctor who can cure "a bad leg," where another practitioner has failed, is in a very fair way for having—many more of them to try his hands on. Sheet-lead is at present the *Coryphœus* among the remedies for ulcers on the Continent, and MM. Roux and Larrey have been giving it an extensive trial.

Two soldiers were admitted under the latter, into the Hôpital de la Garde, with callous ulcers in the legs. The surface of the sores was of a dirty brown, and their edges raised and indurated. After the limbs had been kept quiet for a day or two, and the inflammatory appearance had subsided, a layer of thin and flexible sheet-lead was applied to the surface of the sore, and fixed there by a bandage tolerably tight. In one case the method failed entirely, in the other, the ulcer diminished in size, the callous edges disappeared, and the granulations reached the level of the surrounding skin. Here, however, the remedy had no longer a favourable action, the ulcer continued pale and indolent, and the progress of the cure was completely stopped. Other trials were attended with the same result, and it appeared that, whilst the ulcer was callous with elevated edges, the sheet-lead was of the greatest service, removing the hardened borders, and bringing the surface of the sore to the level of the skin around. When this effect is once obtained, the lead should be discontinued, as it allows of no escape to the pus which is secreted, and prevents the cicatrization of the ulcer.

---

### GUY'S HOSPITAL.

### 17. Thoracic Diseases.

*Case* 1. A sailor, 38 years of age, who had been exposed to cold, wet, and hunger, for six months, began to complain, seven weeks before admission, of shortness of breath, oppression, and cough. His legs swelled, and he came under Dr. Bright in the following deplorable condition. He could scarcely be induced to go to bed, but stood against a chair, leaning his head on the back of it. In this position he continued four nights in succession, without ever lying down! His legs were much swelled—pulse 120 to 130—respiration 48, and laborious—urine scanty. He was cupped on the margin of the ribs—blistered—bled from the arm—took diuretic medicines, purgatives, &c. but without any material effect, and he died in ten days after his reception in the hospital, 9th March, 1827.

*Dissection.* There was a wash-hand basin full of yellow serum, containing fragments of false membrane, in the right side of the thorax—very little fluid in the left side—two ounces in the pericardium—lungs sound—heart twice its natural size—similunar valves of the aorta greatly diseased, being puckered, thickened, and corrugated, so as to have been unfit for their office. The aorta, about its arch, was also diseased. The liver was indurated, speckled, and inclining to the nutmeg-appearance.

*Remarks.* The above case occurred in Guy's Hospital eight or nine years after auscultation had been made publicly known in this country—and presented one of the finest opportunities for putting into execution some of the most important means of diagnosis. Yet no such attempt appears to have been made. The right side of the chest, wherever the water was present, would have sounded as dull as marble—the other side, as tight as a drum. No respiration would have been heard in the lower part of the one side—in the other, it would have been, most likely, louder than natural. The heart, being twice its natural size, would have beaten over a large space—and the semilunar valves of the aorta being puckered and contracted, the "*bruit de soufflet*" would have been plainly heard. But the ear would not be applied to the chest, lest it should look *quackish*, while the phlebotomist, the cupper, and the house-apothecary, were all set to work, in order to cure a disease that was utterly beyond the reach of art! Can the medical officers of public institutions expect to hold their stations in professional estimation, while they thus contribute to roll back the tide of improvement to its source? If they do, they come to the same sapient conclusion to which the ostrich comes, when he thrusts his head into a bush in order to elude his pursuers. He then sees nobody, and philosophically concludes that nobody sees him!

*Case* 2. A man, 49 years of age, was admitted into Guy's Hospital, on the 7th February, 1827, who, for some weeks previously, had been labouring under severe cough, with occasional hæmoptysis. Three weeks before admission, he observed his ankles swell. His pulse was 100, and very sharp, not at all to be felt in the right wrist. He could not lie down—respiration 44, and laborious—mucous expectoration, with some blood—urine scanty and turbid. Purgatives, diuretics, slight mercurials, cupping, and blisters were applied; but he died in a week after coming into the hospital.

*Dissection.* Countenance apoplectic—very little fluid in the chest—lungs not generally unsound; but in three or four places, "decided effusion of blood had taken place into the substance, forming that appearance which has been called pulmonic apoplexy, with a circumscribed sense of hardness to the feel externally, and nearly a defined margin to the dark red part internally."* The heart was twice its natural size—semilunar valves of the aorta thickened and ossified—whole arch of the aorta dilated and diseased. "But the most striking appearance was in the descending aorta, about two inches below the arch, where a spongy mass of bone filled up nearly the whole opening of the aorta for the length of an inch, and, at the origin of the cœliac artery, the same appearance recurred." The liver was granulated and small—the spleen hard—the kidneys sound.

It is not a little remarkable that life could have been preserved so long under such a serious obstruction to the course of the blood through the aorta. The size of the heart and the congestion of the lungs cannot be wondered at, considering the difficulties under which the central organ must have laboured in performing its function.—Dr. Bright's Reports.

### 18. True Pulmonary Abscess.

Dr. Bright, in his recent and splendid work, has only adduced one well-marked case of that rare termination of pulmonic inflammation—abscess. We shall lay this case—or rather the dissection, before our readers.

A man, aged about 30 years, was admitted under the surgeons of Guy's Hospital on account of stricture. On the day of reception, however, he was seized with pneumonia, for which the usual means are supposed to have been employed. One month afterwards, Dr. Bright was desired to see him, but he was dying.

*Dissection.* The right lung was sound. The general substance of the left lung was solid, its colour grey. "A large irregular cavity was formed in the middle lobe, undefined by any distinct cyst, such as is usually seen in true tubercular phthisis, though there had been an effort in the vessels of the surrounding parts to throw out fibrin in such a manner as to prevent the indefinite extension of the suppuration. The cavity had, in part, emptied itself into a large branch of the bronchial tube. The pus which remained was very offensive, and of a dark colour." In no part of the lung was there any tubercular deposit.

We admit that this was an instance of pulmonary abscess—but we contend that it is a very rare termination of the disease. Such an abscess, perhaps, on a somewhat smaller scale, might heal, after a large discharge of purulent matter, and after exhibiting the phenomena of hectic fever, with emaciation and other symptoms of phthisis. Such cases are, doubtless, some of those where phthisis has been said to be cured.

### 19. College of Physicians *versus* Dr. Harrison.

This long expected and much bruited trial at last came on, in the Court of King's Bench, on Thursday, the 3d of July. We do not think that either of the parties concerned has been a gainer in a *moral* point of view—while both of them will be decided losers. The question divided itself into two points of dispute—the validity and meaning of the charter, or the bye-law founded on it—and the proofs of actual practice, on the part of Dr. Harrison. Sir J. Scarlet expatiated very *costively* indeed on the *policy* or expediency of the law which he was instructed to enforce. In fact, he begged the jury to keep the *policy* of the law entirely out of sight, and direct their attention to nothing but the *fact* of Dr. Harrison's having practised as a physician. The *speech* of the College coun-

* These were the portions of lung from which the hæmoptysis proceeded.—*Ed.*

sel, in short, did not consist of much more than twenty or thirty words. But there was one passage in it which deserves commemoration. Sir James observed, that the law made no distinction between the most learned and talented (even the President of the College) and the most ignorant and empirical. All who did not conform to the law were punishable—and justly might the College be accused of partiality if it suffered some to escape, while it put the said law in operation against others!

Dr. Harrison's counsel took an exception, in limine, against the validity of the Charter, as not being confirmed by Parliament, and the point was reserved to be argued before the judges.* The proof—or rather the culpability of practice, then went to the jury. The principal witness of practice was a maid servant belonging to a young lady, with a crooked spine—and well might the patient curse the day that ever she employed a spine-doctor! The cross-examination of the duenna brought out all the secrets of spinalogy—the thumbing, shampooing, stretching of the poor young lady's back, &c. In addition to medicines which were prescribed in aid of the manipulations, it was sworn, that Dr. Harrison prescribed medicine for the maid, while he was attending on the mistress. An apothecary also proved that he made up some of the prescriptions, which were *internal* medicines, and in the hand-writing of Dr. Harrison. These were the proofs of practice, as far as witnesses were concerned; but Dr. Harrison's letters to the College, as published in all the journals of the day, rose in judgment against the writer, and while they proved practice, they certainly made him cut a figure that was any thing but enviable, as will be presently seen.

Then came the defence—and a magnanimous one it was! This defence consisted in pleading that Dr. Harrison only practised surgery! As far as the lady with the crooked spine was concerned, the counsel certainly made good his point but failed in respect to the maid. The sheaf of prescriptions, however, which were produced and sworn to in Court—the evidence of the maid—and, above all, the letters of the defendant himself, formed a chain of evidence, as clear as the sun at noon-day, that Dr. Harrison had practised physic, *as a physician*, for nine or ten years past. This was the decided opinion of the judge—and it must have been that of every individual within the walls of the court, jury and all. Yet what was the verdict? FOR THE DEFENDANT!!

We shall now make a few comments on this extraordinary trial. Our readers are aware that this is the *only* journal which has strenuously resisted the procedure of the College against the regular graduates of regular Universities.* But, as men's memories, now-a-days are very short, we shall take the liberty of refreshing them by a few quotations from former articles.

FIRST EXTRACT.

"This fining and imprisonment, after failing in an examination, shews the difference between former and present times. What would have been the consequence if Dr. Armstrong and Dr. John Mason Good had been *fined and imprisoned* immediately after their rejection by the present College!

"Tempora mutantur, et nos mutamur in illis."

"The College ought to bear in mind how dangerous a procedure it is to kindle up the exercise of Statutes at which science and sense *should* blush—and for which the College itself *may* perchance grieve!"—*No.* 14, *Oct.* 1827, *p.* 517.

SECOND EXTRACT.

"For our own parts, had we been in Dr. Harrison's place, we should, after what had passed, have given up the admission of practice, in order to bring the question to a legal issue. The only reasonable excuse for withholding this admission, is, to try whether the College

* We were surprised to find it stated, in the Medical Gazette, that the point of law was overruled by the Judge. We heard him distinctly say that he would reserve it for the court above. Mr. Campbell was prophetic in his opinion that it would never be argued there.—*Ed.*

* We say the graduates—or rather the *diplomas* of regular universities—for the character or conduct of the individual possessing them has nothing to do with the justice or injustice of the procedure.

can bring home the *necessary legal proof of practice*, against any man. As the prosecution must hinge entirely on the acts growing out of the charter of Henry VIII. and as it is there decided that a man must practise (de die in diem, of course) for the space of a month, before he is finable, so we apprehend that it would be extremely difficult to bring home such proof against Sir Henry Halford himself. If we were on the jury, (for a jury and not a judge must decide as to the proof of Dr. H's practice,) we should certainly interpret the statute as '*uninterrupted* practice for 28 days'—and it is just possible, whatever the lawyers may opine, that a jury will look at the act or statute with the eye of common sense, and not under the influence of corporate pride or legal quibbling."—*No.* 15, *Jan.* 1828, *p.* 248.

After the foregoing extracts, it is hardly necessary to say that we have been tolerably fortunate in our *prognoses*. We appeal to every man in court—and to the judge on the bench, whether it was not clearly proved, by every species of evidence, including the written confession of the defendant, that Dr. Harrison had practised as a physician in this metropolis for ten years past—AND YET A BRITISH JURY DECIDED THAT SUCH PRACTICE WAS LEGAL. What is the oath administered to the jury? It is this:—"You shall a true and proper verdict find, *according to the evidence*, so help you God." We entirely agree with the learned judge, in his charge, that the evidence, including the defendant's own letters, was totally at variance with the defence set up, viz. "that his practice was surgical;"—therefore the verdict was apparently founded on the injustice of the claims of the College to prosecute and fine a man for prescribing an internal medicine, and signing the initials of his name to the prescription. Volumes of these prescriptions and living witnesses proved this kind of practice—but the jury would not levy a fine for such practice. This, we should think, will set the question at rest. It is quite impossible that the President of the College of Physicians can ever again expose that body to ridicule, by coming into Court with an antiquated Charter of Henry VIII. or rather of his monkish minister. One of the extracts above quoted will shew that we predicted the sentiments of a jury on the attempt to enforce laws which violated common sense.

But what shall we say to the defendant? He has covered himself—not with laurels, but with disgrace. After all the blustering and bragging about affording the College every facility to try the *legality* of their Charter, he first withheld every proof or admission of practice—and when these were wrung from him, and found in his own letters, as well as among his own patients, he skulked out of the contest, and *attempted* to prove himself a surgeon! Proh pudor! We say *attempted*—for in this he failed. He did all in his power to leave the question *worse* than he found it. For had he baffled the College merely by proving an *alibi*—by shewing that he was a physician only by name, while he was a surgeon in fact, what would he have done for his alma mater—for the graduates of regular universities, by such procedure? The question is easily answered. He would have left them completely at the mercy of that law which he stood forth, in big words, to abrogate! In short, we have not words to express our contempt for this tergiversation. But as the Jury were sworn, not to return a verdict according to the line of defence, but according to the *evidence*;—and as this proved Dr. Harrison to have practised as a physician, in spite of his shuffling; so the verdict of the jury may serve as an ominous warning never again to attempt the levy of a fine on any regularly educated medical practitioner. We cannot but entertain some faint hope that this trial will open the eyes of the College to the danger of the path which they are pursuing—to the impolicy of *compelling* to the licence, when they have it in their power to render the licence an honour, and consequently an object that would be eagerly sought after. Let not the ears of the public be shocked, and the feelings of the profession outraged by prosecutions instituted against the diplomas of our best medical universities, while quacks of every description are suffered to overrun the land.*

* This trial shews that the College has now no power over practitioners who make any pretence to surgery. The judge indeed observed, that if a man practised

As for Dr. Harrison—he has amply verified the sentiment which we expressed in our last number (p. 88) when reviewing his book. "Now, in our humble opinion, this 'bolder flight' is like that of ICARUS—a complete descent. When he (Dr. H.) soars, on Icarian wings, to the professional public, the wax dissolves—and down he goes." Like a blustering bravado, this modern reformer challenged the College into open combat; but when the College met him fairly and honourably (however impoliticly) in the field, he slunk away, like another Whitelock, with a "white feather in his tail."*

---

20. PARALYSIS CURED BY LIGHTNING.

A vessel, while pursuing her course across the Atlantic, was struck several times with lightning, and the men were strongly electrified. Among the passengers was a man who had been paralytic of both lower extremities for more than three years. He was in bed when the electric discharge took place, and his ship-mates were not a little astonished to see him jump out of his cot and march upon deck, where he continued to walk about as brisk as any of them. The cure was not temporary; for he has enjoyed the use of his limbs ever since. We have lately seen some remarkable effects produced by strong shocks of electricity thrown along the nerves leading to paralyzed muscles:—also in torpid states of the liver, when the galvanic fluid was thrown through that organ.

---

21. SIR MATTHEW TIERNEY'S POWDER.

We do not see why a TIERNEY should not have his powder as well as a JAMES or a DOVER. This is the age, and this is the country for nostrums—and we see much more interest excited by the announcement of one specific remedy than of twenty general principles. A man tells us "arsenic will cure an ague." This is easily understood by all capacities. The name of the disease is only to be ascertained, and any chemist can supply the medicine. But, if the varying forms of the malady—its stages—and the temperament of the subject, are to be studied—that entirely alters the case! The empirical road is by far the pleasantest to travel. The lovers of nostrums will be delighted to hear that we can furnish them with a powder which will make their fortunes. It is a panacea for almost every ill that flesh is heir to—it removes all obstructions—and will ultimately supersede every other kind of aperient. It is indeed a kind of LOVE-POWDER—since we are assured that whoever takes it once, will be so much in love with its operation, that he or she will never afterwards relinquish its use. The following is the formula:—

℞. Sulphatis sodæ.. ℥iss.
Carb. magnesiæ, ℥ss.
Sulph. magnesiæ, ℥ij.

The two sulphates are to be dried in an oven, till all the water of crystallization is completely evaporated—and then the whole three are to be well rubbed together, and kept in a stoppered bottle. The dose is one or two drachms, in a draught, or in a tumbler of warm or cold water early in the morning—repeating the dose, if necessary. Its operation is very mild and pleasant, and we recommend this powder to be kept as a very useful extemporaneous formula.

---

surgery *and physic*, he was liable to be prosecuted by the College. But of what use is the exhortation of the judge? The jury take the liberty of thinking for themselves, and their verdict was in the very teeth of the Bench. It would be far better for the College to apply to the Legislature for a new Charter with ample powers—first securing the cordial co-operation of the profession by a liberalization of its bye-laws.

* It is probable that this trial will give rise to more consequences than are, at present, suspected. The doctrine that all diseases are of *local* origin, is rapidly gaining ground; and consequently, the difficulty of distinguishing *medical* from *surgical* cases will be totally impracticable. Let the College look to this. Let them establish their claims and their distinctions on science, sense, and professional feeling—not on antiquated laws, against which every liberal mind must revolt! We are not the flatterers, the SYCOPHANTS of the College—and, consequently, we may be deemed its ENEMY. TIME will tell who are the REAL friends or the REAL enemies!

22. OBSERVATIONS ON DEPOSITIONS OF PUS AND LYMPH OCCURRING IN THE LUNGS AND OTHER VISCERA, AFTER INJURIES OF DIFFERENT PARTS OF THE BODY. By THOMAS ROSE, Esq. Surgeon to St. George's Hospital.

[Med.-Chir. Trans.]

Ever since the days of Morgagni, and, indeed, long before his time, it was observed that abscesses, or at least collections of matter, occasionally took place in certain viscera and cavities after injuries of the head. Morgagni combats the idea, long entertained previous to his time, that matter descends from the head to the liver only in such cases. Both Valsalva and Morgagni record instances where the lungs and cavities of the thorax and abdomen were the parts affected by purulent depôts, after blows on the head. The Memoirs of the Royal Academy of Surgery contain several papers, by Quesnay, Bertrandi, Andouillé, and others, on the subject of hepatic abscesses succeeding cranial injuries—and Desault considered it a very common occurrence—much more common than it has been found in this country. Desault regards these hepatic abscesses as resulting from the disturbance excited in the nervous system; while Richerand endeavours to shew that they must depend upon some injury sustained by the liver at the time of the accident. But, whether hepatic abscesses happen as frequently as the French surgeons have represented, or not, it is certain that purulent collections in different parts of the body are not unfrequent after injuries of the head and other parts—nay, after operations, however carefully performed. The author of the present paper (Mr. Rose) met with several instances of their occurrence, in the lungs particularly, during the Peninsular War, after amputations and wounds of the extremities. The same has been observed by some of the French surgeons, especially Baron Larrey, who has related the case of General Caffarilli, who died on the 19th day after amputation of the arm. Dr. Hennen has related some interesting cases of this kind in his work on Military Surgery—two of abscess in the lungs, and one in the liver.

But we need not multiply the instances any farther, as the fact has now been well known for some centuries past, though the pathological writers of this country have taken little or no notice of such an occurrence.

Mr. Rose has not been able to connect these visceral abscesses after injuries with any peculiarity of constitution, nor with any peculiarity of treatment. They have occurred when the most vigorous depletion had been pursued in young subjects—and where nourishment had been properly supplied in more advanced age. In all the cases which he has seen, these abscesses took place at some period between the end of the second and that of the fifth week after the accident. In respect to the theory of these purulent formations, Mr. Rose thinks that the explanation of Desault, above noticed, is the most rational. "They are to be classed amongst the effects of constitutional irritation arising from local injury, and are certainly striking illustrations of the irregular action in the vascular system to which that irritation may give rise."

It is not very uncommon to find inflammation or congestion in particular organs immediately after the constitution has rallied from the shock of a severe accident or surgical operation, attended with well-marked symptoms of inflammation. But the visceral affections referred to in this paper have a peculiar character, connected, our author thinks, with the *rapid formation* of pus or lymph in the parts affected. The period of this formation is that in which suppuration takes place in the injured parts, especially if great constitutional injury has preceded. These purulent depôts are often unsuspected, till they are discovered on dissection. The presence or the formation of matter, however, is sometimes indicated by rigors, and other symptoms of suppurative fever, at a time when the wound itself is disposed to heal.

The appearances *post mortem* are remarkable and not easily described in words.

"The disease consists, apparently, of depositions in the cellular texture of the affected organ, partly of a white or yellowish coloured lymph, and partly of pus. These depositions vary in size from beyond the bulk of the largest walnut to something less than a common pea. Where the lymph is most abundant, they may be described as a soft white tubercle of irregular shape, not contained in a cyst, but imbedded in the cellular substance of the part, and gradually blending with its natural structure. When pressed, some pus exudes from them. Where the pus collects in greater quantity, it is lodged in an irregular cavity, probably in the middle of some of the tubercles, and the walls of the abscess are formed of flakes of lymph. The number of these tubercles and abscesses vary in different instances, there being sometimes only one or two, and sometimes the whole viscus being filled with them. In the lungs they are chiefly formed in the parts adjacent to the pleura pulmonalis, and there is often at the same time an effusion into the cavity of that membrane of a sero-purulent fluid mixed with lymph. In the liver and spleen they are dispersed throughout the substance, sometimes shewing themselves in one or more yellowish patches, not elevated, on the convex surface of the great lobe of the former viscus, and at other times lodged in its substance. The parts adjacent to them shew evident marks of increased vascularity." 266.

Mr. Rose does not enter on the treatment, having little to suggest on that head. The reduction of vascular action and tranquillization of the nervous system are the obvious indications—more easily pursued than fulfilled! When the abscesses are once formed, they will be found, as Desault long ago observed, to be almost invariably fatal. Four cases in illustration are related by Mr. Rose, and two by Mr. Lawrence. We shall glance at some of these.

1. The first was a soldier, who was shot through the elbow-joint at the storming of St. Sebastians, in 1813. The inflammatory symptoms in the arm did not run very high, but there was a good deal of irritative fever. After three weeks' attempt to save the arm, Mr. R. was obliged to amputate. On the second morning after the operation, the patient had a rigor and a perspiration, which returned next day. The constitutional symptoms now increased, and he expired on the seventh day after the operation, having exhibited symptoms of oppression about the chest till the last. There was more than a pint of sero-purulent effusion in the left side of the thorax, and the pleura highly inflamed. Numerous circumscribed abscesses were found in the cellular structure of the lungs, but perfectly distinct from the parenchyma, by which they were surrounded. They were not invested with a cyst, and some of them appeared to consist of lymph.

2. This was a case of compound fracture of the leg, admitted into St. George's Hospital in July, 1823. Considerable inflammation and tumefaction supervened, together with erysipelas, which spread over the thigh and abdomen. Deep incisions were made—venesection was employed, and the symptoms subsided; he was seized with a rigor—his tongue became dry and brown, with hot skin, and pain in the epigastrium. He got better for a time, then relapsed, and died in a state of stupor on the 20th day from the date of the accident. On dissection, there was found some serum in the ventricles of the brain—several circumscribed abscesses in the lungs—and, on the convex surface of the liver, a large mass of a perfectly white colour, under the peritoneal surface, being two or three inches in diameter, and not at all blended with the neighbouring parenchymatous substance of the liver, which was sound. This mass consisted of loose lymph, with pus diffused through it. There was another similar mass in another part of the liver. It is not quite clear that these depositions were produced during the period that succeeded the accident, though it is not impossible.

3. Two or three interesting cases are added by Mr. Lawrence—one occurring after an operation for the removal of a cartilage from the knee-joint, and where a great number of small, yellow, elevated spots were observed on the convex surface of the liver, which discharged a thin, purulent fluid when cut into, leaving a yellow fibrous substance, like the flakes of a scrofulous abscess. These depositions varied in size, from that of a pea to that of a hazel-nut, and were several hundred in number.

---

### 23. Chloride of Lime.

Mr. Docker, surgeon of the Windsor Castle, East Indiaman, has made a report on the effects of this important agent in the destruction of unwholesome effluvia between the decks of ships. As the vessel approached the tropics, they sprinkled the orlop decks with the solution, twice a week, and they found that it completely succeeded in removing the disagreeable close smell which always prevails when a number of persons are confined in a limited space, and without a free circulation of air. Mr. Docker attributes a great deal of the good health of the crew and passengers to the agency of the chloride.

During their stay at Saugur Island, in the mouth of the Ganges, the gun-deck was regularly sprinkled, morning and evening, with the solution. The consequence (he thinks) was a comparative immunity from cholera, which was raging fatally in the other ships. The wind blew from the shore all the time, and was loaded with morbific miasmata. In China they used the chloride, and escaped the dysentery which prevailed in the river Tigris at the time. Mr. Docker found the solution of incalculable value in removing the putrid and disagreeable smells that are usually generated in the sick-berths of ships. He also verified the efficacy of the solution, as a gargle, in arresting mercurial ptyalism. In a case of sloughing wound, where all the ordinary remedies had failed, "it speedily put a stop to the sloughing process, and induced the sore to take on a healthy appearance."

We think there can be no doubt that this destroyer or correcter of putrid effluvia will be of great service in the navy, and form a valuable addition to hygiene in general.

---

### 24. On the Employment of Lunar Caustic in Plastic Inflammations of the Mucous Membrane of the Mouth, Nares, Throat, &c.

The mucous membrane lining the above conduits, and also that lining the larynx and trachea, is very subject to inflammation, and this inflammation is often accompanied by an exudation which becomes converted into a kind of false membrane. To this affection Laennec and some others have applied the term *plastic*. It is often epidemic—sometimes sporadic. But the membranes above-mentioned are subject to a chronic inflammation, of a passive kind, in which the vessels are enlarged, their secretions increased and vitiated, and the patient kept in a very uncomfortable state for years. The uvula frequently becomes relaxed and elongated, and by tickling or irritating the epiglottis, keeps up a hacking cough, which excites great alarm in the mind of patient and friends. It is in this complaint, a very prevalent one, that an eminent surgeon of this metropolis has gained great reputation by employing lunar caustic in solution, on a sponge or brush, and very freely applied to all the above parts, as far as they can be reached.

A French physician [M. Gerouard] lately transmitted a memoir on this remedy to the SOCIETY of MEDICINE, in which he relates many cases of its success in the plastic inflammation alluded to, and recommends the caustic to be applied, not only to the fauces, nares, and parts adjacent, but to be introduced into the larynx, where the inflammation has affected that part. M. Gerouard frequently employs the nitrate in substance, carefully fixed in a silver tube, straight or crooked. He observes, that to cause the false membranes to be detached from the larynx or trachea, it is not always necessary that the caustic should enter these conduits. "It is sufficient, in many cases, to touch the superior aperture. I have seen patients eject membranous tubes, of some inches in length, the day after the rima glottidis was touched with caustic." Dr. Authenac has also laid before the public some cases where the lunar caustic proved very beneficial. Dr. Brown, of New York, has also published a paper on the same subject, in the January Number of the Medical Recorder for the present year.

But we wish to draw the attention of the profession to the employment of this topical application, in the chronic inflammations and relaxations above-mentioned, as well as in laryngeal affections simulating phthisis. We have seen some cases recently where chronic coughs, accompanied by muco-purulent expectoration, that had harrassed patients for years, and baffled all their physicians, gave way, in a very rapid manner, to a few applications of solution of lunar caustic on the sponge of a common probang. These hints may prove useful to many of our brethren. The state of the fauces should be carefully examined in all cases of chronic cough; and the epiglottis may be often seen by pressing down the tongue. That part, and even the rima glottidis may be always examined with the finger.

---

### 25. Vapour Bath.

It is not our intention to dilate on this important remedial agent. Its powers are every day becoming more apparent. But we believe that the utility of the vapour bath may be much increased by every contrivance which adds to the facility of distributing the vapour with different degrees of intensity to different parts of the body, to which it may be desirable to induce what is called a determination of blood. Our ideas will be more easily understood by a short statement of facts.

Captain Jekyll, of the Royal Navy, having been long a sufferer from rheumatic gout, was advised to try the hot baths at Bath. But each bathing excited fever and head-ache. The vapour-baths were then resorted to; but he found a great inconvenience attendant on their being much hotter at the upper part than at the feet. They consequently gave him a violent head-ache—his gout left the feet, and caused excruciating spasms in his stomach.

"Finding" says Captain J. "that steam removed the gout to that part where the heat was the greatest, I was convinced, that by reversing the plan, and making the feet much hotter than the upper part, the gout would go from the stomach to the feet again: consequently, I invented a vapour bath which happily produced that desirable effect, and immediately removed the gout from the stomach to the feet, relieved the pain, and promoted a comfortable sleep for some hours; and so effectually subdued the gout, that in two days I was perfectly recovered. I have since, on several occasions, experienced the correctness of my system, of making my vapour bath ten or twenty degrees hotter at the feet (by Fahrenheit's thermometer) than at the upper part of the bath; which is easily regulated by the person taking the bath, or by an assistant. The gout descended to the feet; where, the pores being opened, a profuse perspiration took place, and those paroxysms, that so often confined me to a bed several weeks at a time, have since yielded to steam with very little pain, and scarcely one restless night in about as many days as it previously continued weeks."

The bath which Captain Jekyll has constructed, is extremely simple, and easily managed, as we can testify from seeing it in operation. It has generally a calico covering; but an oil-silk one is preferable, as it confines the steam much better. The dress is kept off the shoulders by a hoop and three supporters, and the whole apparatus packs in a small box, and costs but a few pounds. It is put in operation in the course of a few minutes by merely putting the boiler on the fire, and placing the patient in a chair on the floor. This portable bath may be seen at 38, Warwick Street, Golden Square. We conceive that it will prove a valuable apparatus, in a small bulk, in our fleets, armies, and colonies, as well as at home. The various purposes to which it may be applied, will readily suggest themselves to our medical readers.

P. S. The following case, which we have just perused in a late number of the Revue Medicale, will be found to bear on the subject now under discussion.

*Case.* The Subject was no less than a Prince—the Prince of Wagram, a personage who figured on the political stage during the reign of Napoleon. He is now in his 62nd year, of robust constitution, but subject, for many years past to annual attacks of gout, which have latterly increased in intensity. The feet were the domiciles of the disease, and the Prince had recourse to patience, in general, for his cure. The year before the fatal campaign of Moscow, the prince was attacked with an unusually severe fit, and the fever and inflammation ran so high, that he was obliged to be bled and considerably reduced. The almost superhuman fatigues of the Russian expedition and retreat were attended with one apparent advantage—escape from the Autumnal attack. But whether it was this escape, or the privations of body and anxieties of mind that affected the prince's health, it so happened that he reached Posen in a very deplorable condition, and it was at this time that M. Mestivier was called to him. He found the prince in the greatest anguish which it is possible to imagine—the face and body quite yellow—the countenance sunk—the lips dry and pale—tongue coated yellow—thirst urgent—hiccup constant—breathing short and difficult—no cough or palpitation—the epigastric region so tender and painful that he could not bear the weight of the bed-clothes. This pain occasionally stretched towards the right hypochondrium—there was no passage through the bowels for three days—urine scanty and red—pulse small and quick. Dr. Mestivier, as well as the physician in attendance, conceived the case to be gall-stones, and prescribed castor oil and purgative lavements. The oil was vomited, but the bowels were opened most copiously. No amelioration of the pain took place—it was inexpressible, and debility was increasing. The physicians now began to suspect that gout had fixed its residence in the stomach, instead of the extremities, and sinapisms were applied to the feet, after hot pediluvia. The night was passed without sleep, and in dreadful sufferings; but next morning the feet became the seat of great inflammation from the sinapisms, and one of them was evidently affected with gout. The pain in the epigastrium vanished, and the gouty foot went through the regular stages without any sinister accident.

Cases analogous to the above are not unfrequent, and in such circumstances we have little doubt that the vapour bath directed to the extremities would very rapidly relieve the stomach, brain, heart, or other part labouring under the retroceded or misplaced gout.

---

### 26. Poisoning by Prussic Acid.

The horrible catastrophe which happened a few weeks ago in the Bicêtre, is known to our readers. The following mistake was the cause of the fatal accident. M. Ferrus, a talented physician, was in the habit of employing prussic acid in epileptic cases, and the formula which he employed was that given by Magendie, under the title of syrup of hydrocyanic acid, which consists of one part of "medicinal prussic acid" to 128 parts of clarified syrup. This medicine, when prescribed in private practice, was always procured at the shop of M. Pelletier, and M. Ferrus was in the habit of giving an ounce of the syrup for a dose, with good effects. This dose contained not quite five grains by weight, of the medicinal prussic acid. M. Ferrus wishing to try the medicine on a large scale at the Bicetre, ordered it in the dose of an ounce, to 14 epileptics. The house surgeon observed at the time, that they never ventured on more than a drachm of the syrup in the 24 hours—and that, even in this dose, considerable effects were frequently seen to result. This observation induced M. Ferrus to order only half an ounce, although he was confident, from repeated experience, that double the quantity would be borne with safety. The medicine was accordingly procured from the "Pharmacie Centrale des Hôpitaux," where, unfortunately the syrup was prepared according to the Codex, in the following manner:—

℞ Syrupi Simplicis partes 9.
Acidi Hydrocyanici p. 1. misce.

By this form, the dose (℥js) contained upwards of 57 grains of the prussic acid!! The infirmary man commenced the exhibition of the medicine on the 14 epileptics. By the time he had given it to the seventh patient, the first was dead. In less than 20 minutes the whole of the seven expired! Magendie had long ago condemned the formula given in the National Codex. The following are his words, and unfortunately they have proved but too prophetic!

"Cet syrop, ainsi preparé, ne pourrait être administré que par gouttes. Si par malheur, une once etait mêlée dans une potion, il en resultera un breuvage mortel."

We fear that M. Ferrus will not escape without some censure. It surely is the duty of the physician or surgeon to know the composition and strength of medicines as directed by the National Pharmacopœia; and if he prescribes according to other formulæ than those contained in the said pharmacopœias, he should explicitly point out these extra-officinal forms for the guidance of the compounder of his prescriptions.

M. Gendrin, the editor of the "JOURNAL GENERALE DE MEDICINE," has made some severe remarks on the ignorance displayed in the National Codex, and argues strongly for the abolition of all national pharmacopœias. He maintains that, instead of leading to uniformity of composition, they produce a diametrically opposite effect. Every chemist goes by his own formula, and the practitioner is daily and hourly deceived by prescribing according to a Pharmacopœia which is hardly ever followed. In this sentiment we cannot entirely agree with our Parisian confrere. We know, indeed, that the evil of which he complains, exists; but we do not think it would be remedied by the abolition of a national or standard Pharmacopœia, however imperfect that standard may be. We are of opinion that, where any potent, and consequently poisonous substance is prescribed, it ought to be done so in an extemporaneous form. For example, the prussic acid should be prescribed, not in a formula where it only makes a part, as in the above syrups; but in draughts or mixtures, where the quantity of the acid is distinctly specified.

The bodies of the seven epileptics poisoned by the prussic acid, were examined by Messrs. Adilon and Marjolin. There was no physical lesion discovered to account for the epilepsy. The hearts were empty, and the blood was fluid in the vessels. It is curious that the bodies exhaled no odour of prussic acid.

---

### 27. ON THE CATARRHUS ÆSTIVUS, OR SUMMER CATARRH. By J. BOSTOCK, M.D. &c.

[Med. Chir. Trans. Vol. XIV.]

In the tenth volume of the Medico-Chirurgical Transactions, Dr. Bostock gave some account of a disease which he conceives to be of "a specific nature," occurring only at a certain period of the year, and from which the author himself had suffered. In short, it was the *hay-fever*, of which we have heard so much, for fifteen or twenty years past, and to which Royalty itself, as well as Dr. Bostock, is subject.* Since the publication of Dr. B's paper, he has seen or received accounts of twenty-eight cases—eighteen of which were distinctly, and ten less imperfectly detailed. They all agree as to the season of year in which the complaint occurs—the seat of the disorder (mucous membrane of nose, fauces, and "vesicles of the lungs")—and the exciting cause.

Except a short observation of Dr. Heberden's, our author has not been able to find any account of the disorder in any work, ancient or modern. In 1822, Dr. Baillie informed Dr. Bostock that he had seen three cases similar to his own. Sir Henry Halford could have given him at least one case, as early as 1814, at which period the writer of this article transmitted to Sir Henry an account of the disorder as it occurred in an exalted personage of the present day. Be this as it may, it appears that, in the twenty-eight cases above-mentioned, the complaint commenced about the end of May or the beginning of June, and continued from four to eight weeks.

"Most of them are attended with fulness of the head, stoppage of the nose, sneezing, watering of the eyes, and discharge from the nostrils. In about half of the whole number the respiration is considerably affected, and in three or four instances it is almost the only symptom. Some of the cases are attended with distinct cough, most of them with irritation of the fauces, and some with a degree of sore throat. Actual inflammation of the eyes is not a very common occurrence, and in some of the cases there is not even the discharge of tears, or the irritation of the eyes. The degree of general indisposition varies very much in the different cases; in some, the patient, during the whole period, is unable to use any exertion, or to continue his ordinary occupations, while, in other instances, he feels no inconvenience, except what arises from the fits of sneezing, and the copious discharge from the nose." 439.

No decided connexion between this

---

* His Royal Highness the Duke of Clarence suffered from this complaint for a great many years. It occurred, in May or June, at the period of cutting down the hay.—*Ed.*

disorder and age, sex, constitution, or mode of life, could be traced. In no instance, has the liability to it been lost by time—all the cases occurred in the middle or upper classes of society—a majority of them were males. It appears to occur equally in town and country.* The proximate cause, or rather condition, is obviously an affection of the mucous membrane of the parts already mentioned. Dr. B. divides the disease into four varieties according as the eyes, the nose, the fauces, or the lungs are affected. It is only in this last variety that Dr. B. has observed the constitutional symptoms of fever and subsequent debility in any considerable degree :—

"And in this case I think we may account for the effect, by supposing that the thickened state of the membrane which lines the vesicles, prevents the oxygen of the inspired air from duly acting on the blood." 442.

The above passage, short as it is, will shew how men are warped by theory and prepossession! Dr. Bostock is a great chemist. The hay-fever is therefore a deficiency of oxygen in the blood! Alas! Poor Physic! No sooner does a man of genius start on a journey of investigation, than he begins to curvet, from side to side, in pursuit of the apples of Hippomenes—or rather of hypothesis, by which he generally loses the race! Dr. B. sets out with the proposition that the vessels of the mucous membrane are in a state of "*increased action*," and therefore, we suppose, they have so much other work on hand, that they cannot attend to the absorption of oxygen or the elimination of carbon! Has it never occurred to Dr. Bostock that inflammation of the mucous membrane of the stomach or bowels will cause fever and other constitutional symptoms? Is this fever—are these constitutional symptoms the result of defective oxygenation of the blood?

But let us turn to the exciting cause. The popular opinion is, as we observed above, that the effluvia of the newly-cut hay give origin to the disease. Dr. B. denies this, as far, at least, as his own case is concerned. Thus he made choice of Ramsgate as his residence, during the summers of 1824-5-6. The last two of the above years were remarkable for their heat; but by procuring a house on the cliff, exposed to the German Ocean, and commanding complete ventilation, he nearly escaped the disease in 1825. In the next year he had some severe paroxysms, though they were mitigated by the coolness of the situation. As there is "not an acre of meadow ground in the Isle of Thanet," Dr. B. thinks it must be the inevitable conclusion that the hay had nothing to do with the disease. We do not agree with Dr. B. respecting the non-existence of an acre of meadow grouud in the Isle of Thanet—and allowing that the hay was capable of producing the disease, we cannot suppose that the Isle of Thanet, or any other spot in England, can be free from the effluvia which are wafted about on every wind that blows. During the Summer of 1827, Dr. B. spent his time at Kew, in the immediate vicinity of meadow grounds. The Summer was very cool, and "except during a few *hot days*, when I suffered *as usual*, my complaint was in a much less degree than the average." The advocates for hay-miasmata would say, that the cool season was unfavourable to the plentiful production of the cause—and when the days were *hot*, the Doctor suffered as usual. But, putting himself out of the question, Dr. B. acknowledges that he has received information from various quarters, "of individuals who have felt no doubt that the complaint was brought on by the effluvium from hay, and was relieved or prevented by avoiding this effluvium." For our own parts, we have little doubt that the complaint is dependent on some exhalation from the surface of the earth, though the precise nature of the miasm may for ever elude our senses.

In respect to the cure or mitigation of the disorder, our author has little to say. Most of the individuals included in the catalogue have tried change of residence, with more or less benefit. In two cases of considerable severity, the patients were better in London than in the country.

* The case to which we have alluded occurred at *sea*—at least, on the coast of France, and therefore far from the immediate effluvium of hay. But it must be recollected that, in the hay-making season, the odour of the drying vegetables is expanded universally through the air of northern countries.—*Ed.*

A third derived benefit from the sea-air. In other instances, no benefit was obtained by change. In respect to medical treatment, Dr. B. is convinced that depletion is injurious, and that some benefit is derived from a moderate use of tonics. On these two points, and on these *only*, do all accounts agree. We think this very circumstance corroborates the supposition that the complaint arises from malaria. Speaking generally, all malarious diseases require tonics sooner or later in the course of their progress. Dr. B. does not expect a cure in his own case. The *habit* has probably become too inveterate.

"Bathing the eyes in tepid water, and fomenting the face generally, occasionally applying small blisters to the chest, mild purgatives, small doses of ipecacuanha, Dover's powder, squills, and digitalis, bathing the feet in warm water, a moderate, but not spare diet, perfect rest, and carefully avoiding all extremes of heat, comprise the whole of the means that I have found useful to myself." 446.

He has been informed by a friend, that great relief was obtained, in two cases, by the application of a weak solution of opium (two drops of the tincture to an ounce of water) to the eyes and nostrils.

---

### 28. ON SOFTENING OF THE STOMACH. By PROFESSOR AUTENRIETH, and DR. CAMERER.

Dr. Camerer, under the direction of Professor Autenrieth, (who may be considered as the real author) has published a thesis on the above subject, which we deem worthy of a short notice in our Journal.

In the first chapter, Dr. C. furnishes us with the symptomatology of the complaint, of which the most important items are, diarrhœa, vomiting, cough, and oppression of the breath, constant restlessness, peculiar expression of suffering in the countenance, perpetual crying and moaning, (in children) drawing up of the legs towards the belly, rapid emaciation, and, towards the end, convulsions, stupor, &c.—Finally, fever, more or less intense, inextinguishable thirst, and complete loss of appetite.

The causes of this dangerous malady are investigated in the second chapter. It is well ascertained that softening of the stomach may take place, without any antecedent disease, and simply as a cadaveric change. In numerous experiments which the authors have made on animals, the great cul de sac of the stomach was found in a state of mollescence, the parietes dissolved and perforated, the extent of this disorganization being in proportion to the length of time that elapsed between death and dissection. But putrefaction did not appear to be at all connected with this change in the stomach. In all cases the stomach had been filled with food at the time of death, and, consequently, there had been an active secretion of the gastric juice, to the solvent properties of which our authors attribute the disorganization of the stomach. In animals who had died of disease, or after a long agony, this species of mollescence was never found—probably (our authors think) from the gastric juice having lost its solvent properties. That the succus gastricus is capable of dissolving the animal tissues, is proved by its action on the neighbouring parts, when perforation of the stomach and extravasation of its contents take place. In these cases, the gastric fluid is found to be of an extraordinary degree of acidity. The authors made some experiments with the liquid found in the stomach of two children dead of gellatinous mollescence of the stomach. A drachm of this fluid was introduced into the stomach of a man recently dead, and the stomach was kept in a temperature of about 50° of Fahr. for 12 hours, when the coats, as far as the peritoneal, were found completely dissolved, wherever the liquid came in contact with them. The same result was obtained in the stomach of a rabbit recently killed. Introduced into the stomach of a living animal, this juice had no effect. The solvent properties of the succus gastricus appear to our authors to be in direct proportion to its acidity—and the acetic acid seems to have the principal share in the mollescence, and in this conclusion the experimenters are corroborated by several others. They are of opinion, however, that this acid will not alone, how concentrated soever, dissolve the coats of a living stomach, unless that stomach be considerably below par in point of vital power. This fact they think they have proved by experiments made on the nerves leading to the digestive organs. The par vagum and grand sympathetic nerves were divided, on both sides, in several animals, and the fluid extracted from the stomachs of children who had died of gastromalacia (mollescence of the stomach) was introduced. In others, acetic acid was introduced, after section of the nerves—and in others still, there was nothing introduced after the nerves were cut. In those animals where the gastric fluids and the acetic acids were introduced, there was found an unequivocal dissolution of the coats of the stomach. The mere section of the nerves was not followed by any such appearance—or at least by very little.

The fourth chapter is dedicated to a consideration of the nature of the disease under review. They conclude that the stomach is enfeebled—almost paralyzed, in this malady, and that there is, moreover, an affection, of an inflammatory nature, of the par vagum. They have been led to this latter conclusion, 1mo. By frequently observing an unusual hardness of the pons varolii and medulla oblongata, on dissection of subjects dead of this disease, the other parts of the brain retaining their natural consistence: and, 2dly, by the almost invariable accompaniment of respiratory disorder in mollescence of the stomach. This inflammation of the pneumo-gastric nerves ends in paralysis of the same nerve, as shewn by cessation of vomiting and of diarrhœa, a little before death. But, as the vascular system is not proportionally impaired, as proved by the continuance of the febrile phenomena, the gastric juice continues to be secreted, and that of an extraordinary acidity, so as to be capable of dissolving the coats of the stomach, already in a weakened state.

Such is the theory (somewhat hypothetical, certainly,) which the authors believe to be conformable to, and supported by, facts. They have adduced 50 cases of this mollescence of the stomach, the greater number of which have been communicated by Dr. Jaeger, and many of them are very interesting. Although we cannot entirely subscribe to the views here taken of the cause of the disease, yet we think enough has been brought forward to prove that an affection of the nerves leading to the stomach may powerfully alter its functions—perhaps even its structure. Thus, melancholy emotions of the mind lessen at once the vital powers of the stomach, through the medium of the nerves, at the very time when this derangement of function produces the most acrid state of the gastric secretions, which tend farther to annoy the organ that gave them birth. This opens out an immense field for practical observation independent of all hypothesis. The thesis of Dr. Camerer, and the views of his master, Professor Autenrieth, are deserving of much consideration.

---

### 29. CASE OF PERFORATION OF THE STOMACH. By CHARLES DAVIS, M.D.

On the 1st of January, 1828, Dr. Davis was called (in conjunction with Mr. R. Carmichael) to Margaret Browne, a servant of Mrs. Bourne's, of Mount-joy Square, Dublin, whom they found labouring under symptoms indicating the action of some acrid poison. She complained of violent pain at the epigastrium, accompanied with retching and vomiting of frothy mucus. She had burning pain along the course of the œsophagus, and most distressing tension and exquisite tenderness of the abdomen. Her pulse was almost imperceptible at the wrist, and her cheeks, lips, and extremities, were cold and livid. She appeared to suffer extreme agony—complained of violent thirst—had hiccup occasionally—and the muscles of the face were agitated with spasmodic twitchings.

Satisfied by the assurances of the family, and the examination of the matters vomited, that her symptoms were not caused by poison; and having ascertained, from an attentive inspection of the abdomen, that neither gastric—hernia, nor any other species of protrusion existed, she was ordered a stimulating terebinthinate enema, and hot salt to be applied to the epigastrium, with bottles of warm water to the feet, but without any hope of advantage, as she was evidently moribund.

The following history of her case was obtained:—She was aged 22 years, of rather full habit, and brilliant complexion;—had hitherto presented the external appearance of good health, but was occasionally heard to complain of pain in the region of the stomach. On the preceding evening, at 11 o'clock, she appeared in the best health and spirits. Shortly before 12, however, she experienced a slight return of the pain in the stomach, for which she bathed her feet.

At five on the following morning she was taken so ill, that the family apothecary was sent for, and he took some blood from her arm, while a bolus, composed of calomel, jalap and opium, was administered, to be followed by an aperient mixture. *She complained that the mixture spread over her belly, and burned her inside.* She continued to grow worse until half-past 12 o'clock next day, at which time she was seen by Dr. Davis and Mr. Carmichael. She soon afterwards expired in convulsions.

*Examination, 27 Hours after Death.* Putrefaction had already set in, to a considerable extent, accompanied with livid patches on the cheeks, abdomen, and thighs. The joints were rigid, and the tension of the abdomen, such as to expose its contents to considerable danger of injury from the knife in opening it. Air, and a turbid, yellowish fluid, were effused in considerable quantity, and the intestines were greatly distended with flatus. An oval opening was found in the stomach, through which the effused fluids had passed. The liver was considerably softened, so as to admit of being very readily broken by the finger.

The intestines were in every respect healthy, as were the peritoneum, spleen, pancreas, and pelvic viscera. The uterus was in a state of incipient menstruation.

The ulcerated opening was upon the anterior surface of the stomach, near its lesser curvature, and pretty nearly midway between its orifices. It seemed as if the coats had been punched out to the size of a sixpence. The edges of the aperture were somewhat thickened, and the peritoneal coat of the stomach, to a small extent around it, was slightly vascular. On the internal surface of the stomach, nearer to the pylorus, were two oval depressions, the edges of which were smoothed down, an appearance which ulcers usually present, when the ulcerative process has extended only through the mucous membrane. In other respects, this membrane *was softer and more pulpy than natural.*

In the great rapidity and violence of the symptoms, and in its fatality within twelve hours from the first accession, this case resembles that published by Dr. Crampton, in the eighth volume of the Medico-Chirurgical Transactions, and most of those cases related by Mr. Travers, in the same work. It differs from them in the larger size of the ulcerated aperture, and in the total absence of any trace of inflammation of the peritoneal surface, excepting about the size of a crown piece, surrounding the perforations. This exemplifies the fact, that inflammation of serous membranes, when it proves fatal with rapidity, by an impression made upon the nervous system, may, when the inspection has been deferred for 24 hours, be totally undiscoverable.

The plumpness, and general appearance of the patient, was such as to lead the medical attendants to suppose chronic disease of the stomach one of the last affections she could labour under. From the first accession of the symptoms, she gave herself up, being firmly impressed with a conviction of the total inutility of medical assistance.

The stomach is amongst the preparations in the Museum of the Royal College of Surgeons.

---

30. Observations on the Effects of the Sun's Rays on the Human Body. By John Davy, M.D. Physician to the Forces.—[Ed. Med. Chir. Trans.]

The sun has been called a universal tanner; and certainly his general action on the surfaces of human bodies, especially in hot climates, entitles him to this appellation. Dr. Davy thinks that the modus operandi of this embrowning process has not been hitherto investigated fully; and, therefore, he seems to have dedicated some of his leisure hours, at Corfu, to the inquiry. Having exposed his naked arm to the ardent gaze of an Ionian Apollo, for an hour and a half, on a hot day in July, Dr. D. found the effect to be an erythema, with pain, and ultimate desquamation of the cuticle, which desquamation continued, more or less, for nearly three weeks, leaving the skin in that brown state, in which it is commonly said to be "when tanned by the sun." This discoloration was not quite effaced at the end of seventeen months.

This being the case, Dr. D. considered himself justified in questioning the accuracy of Dr. Bostock's opinion, that the sun's action was exerted on the epidermis, "because we are informed that the tan of the skin is removed by blisters." Dr. D. observes that the skin, instead of being rendered fairer, is made browner by blisters. He concludes, therefore, that the seat of discoloration is in the surface of the cutis, and not the cuticle or rete mucosum. He has examined, with some attention, the cuticle of the negro, of people of colour, and of Europeans, who have become dark-brown from exposure to the sun between the Tropics: and in each instance, when detached, the said cuticle has appeared much less coloured than the skin. He thinks that the colour, in all these instances, is owing to a kind of pigment deposited in minute particles or filaments, on the surface of the cutis, analogous to the pigmentum nigrum of the eye. Dr. D. doubts the existence of rete mucosum in any instance.

The cause of the solar browning—or rather the modus operandi of the solar rays in darkening the colour of the skin, is next entered on by our ingenious author; but we do not see much that is either conclusive or important in this part of his paper. The *consequences* of the discolouration of the skin are more important. Dr. Davy has verified the experiments published by Sir Everard Home in 1821, shewing that, when the skin is painted black, it is defended from the scorching effect of the sun's rays; but he is doubtful of the correctness of the inference thence drawn, that the black rete mucosum of the negro possesses the same protecting power. The cases, he thinks, are not parallel. In the one, the colouring matter is external to the insensible cuticle—in the other, internal. There are some facts, however, connected with this inquiry, which are interesting. Thus:—"when the sun's rays are concentrated by a lens, they penetrate, I find, through bone, as a portion of the cranium—through nine folds of black crape—and what is most extraordinary, through rolled platinum." This circumstance may help to explain the effect of the sun on the brain, in producing that malady commonly called *coup de soleil*. The result of some curious experiments made by Dr. D. lead him to conclude that, although the colouring matter of the negro's skin affords some protection from the scorching effects of the solar beams, yet that it does not afford all the protection possessed by the negro. We have little doubt indeed that Provident Nature adapts the skin impressed by the sun's rays to bear them afterwards without inconvenience. But there appears to be other means than the mere colour of the skin. Perspiration is one—and there may be *internal* processes which tend to counteract the solar heat.

Whether there be a radical and primary difference between the organization of the African and European we need not now inquire; but we suspect that *habit*—that is, the continued operation of physical causes on the human constitution will account for all the varieties we find in man, when assisted by hereditary transmission.

The following extract is interesting:—

"Within the tropics, when on a journey, I have tried the temperature of my palenkeen bearers, just before setting out, when they were agreeably cool; and I have tried it again after three or four hours' exertion exposed to the sun, at a temperature between 80° and 90°; and I have found their temperature rather diminished than increased, as indicated by a thermometer, the bulb of which was placed under the tongue, or in the axilla.* Whatever occasioned this reduction, or keeping down of animal heat, this I allude to as the internal means of counteracting external heat. However it is explained, (and it would not be difficult to offer a plausible explanation of it,) the fact seems to be curious; and if it is as new to others as it was novel to me, when I first made the observation, I need offer no apology for introducing it."

---

* "Observations which I made many years ago within the tropics, with some care, indicated, that moderate exercise raised the temperature of the internal parts of the body, as well as of the surface; and that severe and long continued exertion rather had a contrary effect, as in the instance above recorded."

### 31. HORÆ LACONICÆ.

### No. I.

To Argus were given 100 eyes; and his task was to watch a single living creature. How many organs of vision would he have required to watch the Press—that confluence of mighty rivers, which, collecting their innumerable tributary streams from every mountain, plain, and valley of literature and science, roll them in countless volumes towards the ocean of oblivion at last! A contemplative philosopher, standing on the banks of this rapid torrent, might amuse himself by calculating what proportion of its waters were expended in fertilizing the soil—what proportion fell into swamps and marshes, apparently for no other purpose than pollution of the air—and what proportion rolled on, unseen, and unheeded, to that vast reservoir, serving at once for its cradle and its grave! Some train of reflections, of this kind, must have been passing through the mind of an esteemed transatlantic contemporary,* when he placed at the head of his periscope, a female figure winnowing corn, or, in other words, separating the grain from the chaff, with this motto underneath:—"Utilia fideliter excerpendium." There could not be a more appropriate head-piece to this department of his Journal. The task of Hercules, in cleansing the Augean stable, was nothing compared with the process of winnowing the medical journals of Europe and America. He had only to let in a river, and sweep the whole rubbish away—but the winnower must darken the air with chaff before he can collect a handful of grain.

Modern ingenuity, and the "march of intellect" were never so distinguished as in the art of adulterating *things* by means of *words*. Medical Reporters, in which we include all species of writers in the periodicals, seem to be as loth to come to the naked fact, as to the point of a naked sword. They are absolutely in a fever, lest the reader should too soon guess at what is ultimately to come out in the course of the paper. Many of the facts, indeed, when developed, are such as prove the writer to have had some degree of cunning, in keeping them so long concealed in verbiage;—but why those who have useful knowledge to impart, should labour to envelop it in obscurity by a profusion of unnecessary words, and thus curtail rather than extend its circulation, we never could divine.

We have been led into this train of reflection by the receipt of a packet from an old but unknown correspondent, Lacon, (now of Albemarle Street) from whom we had not heard for some years, and therefore concluded him to be dead. He informs us that he has been immersed in the most profound studies, during the last five years, and that he has made a discovery of the greatest importance, especially to medical literature.

By means of an elaborate chemical, or rather alchymical process, he has constructed a metallic plate or Tractor, which he denominates a Verbifuge, and which, being drawn lightly over the pages of a book, immediately effaces all *unnecessary* words or sentences, leaving the efficient ones in insulated groups, which often, he says, present a very curious appearance, and one that would be most appalling to authors.

Apparent rari nantes in gurgite vasto!

Perceiving, at a glance, the vast importance of such a tractor to an analytical journalist, we offered a handsome sum for the annual use of the Verbifuge; but he would neither lend nor sell his tractor, though he promises to amuse himself by *deverbatising*, (as he expresses it) certain portions of medical literature, and sending us the nett proceeds. With this our readers, as well as ourselves, must be content.

It is interesting to learn, that Lacon is now devising a text by which he hopes to distinguish truth from falsehood, a task which the Tractor does not pretend to perform—its office being simply that of a Verbifuge. We will venture, in a few instances, to append some remarks to the communications of Lacon; but shall never interfere with his text.

### Verbifugal Operations.

1. *Subjects for Dissection.* Sir George Mackenzie has (he says) discovered the

* American Medical Recorder.

art of embalming, and has extended this useless art to a very useful purpose—"*the preservation of subjects for dissection.*" The dead body being put into a cask, the vessel is to be filled with a composition made of one measure of molasses, one of salt, and 15 or 16 of water. The cask is then to be headed, and two small holes left open for the escape of air, and the introduction of liquid during 24 hours, when the apertures may be closed, and the cask forwarded to its destination. In this way, he has no doubt that subjects may be sent fresh (*sweet*, at all events) from the West Indies or the Coast of Africa, if the casks are kept tight. He has specimens that have been thus preserved four months. Dr. Christison corroborates this statement.

REM. This may prove a very important discovery, if true; and will do more to facilitate the study of anatomy than a committee of M.P's.

2. *Tetanus.* Mr. Alexander (of Liverpool) has lately stated a case of traumatic tetanus in a sailor, where, after amputating the finger that was wounded, thirty ounces of blood were taken from the arm, and then the following medicine exhibited:—

"℞. Tabaci fol... ʒj.
Aq. bullient. ℥iv.
Ft. infusum, capiat dimidium statim, et reliquium post horas tres."

The first dose nauseated the stomach, and produced some relief of the sternal pain. The second dose produced both vomiting and purging, with still greater relief. Two doses more, of the same strength, removed the emprosthotonos.

REM. The quantity of tobacco taken in this case, (two drachms in infusion) is so enormous, that there is some reason to doubt a typographical error. If the fact is as stated, then all that can be said is—"anceps remedium melius est quam nullum."

3. *Hydrophobia cured by Venesection.* LACON writes this heading with fear and trembling; yet the facts appear strong. A boy had been bitten by a dog, whose history could not be made out. Nearly four months elapsed, when unequivocal hydrophobia became developed. Dr. Charles Davis, of Dublin, and several other medical gentlemen, saw the patient. Bleeding, purging, blistering—or perhaps NATURE, dissipated the hydrophobic symptoms; but pneumonia unexpectedly terminated the boy's life on the 14th day after the accession of the hydrophobia. This was very unfortunate. The dissection shewed sufficient cause of death in the lungs; but no physical trace of the hydrophobia was discovered any where.

4. *Aneurism of the Temporal Artery.* Assistant Surgeon Bushe, M. D. is an adept in the noble art of amplification. It is to be hoped that, when he writes a system of surgery, he will emplo LACON to revise the manuscript—otherwise Fort Pitt will not hold the volumes. In three cases of temporal arteriotomy, aneurisms by anastomosis, formed, which resisted the usual means, and ended in fungous bleeding tumours, that were removed by carrying incisions round their bases, and dissecting them away. In two of the cases, the artery had been completely divided; but this did not prevent the aneurism from taking place. In all three, pressure was applied, and seemed to expedite the growth of the tumours, as well as excite constitutional irritation. In one case, a circular incision was made round the base of the tumour—but not carried to the bone, as recommended by Sir Astley Cooper—and this plan failed. How could it be expected to succeed, when the supply of blood from the deep-seated vessels was left undisturbed? The author comes to the concluslon, that excision is the only safe and radical cure—and that it should be performed as soon after the formation of the tumour as possible.

That species of aneurism (by far the most common) in which the trunk of the vessel is the seat of the dilatation, has never occurred, in Dr. B's practice, where proper pressure had been applied after arteriotomy, whether the vessel was cut across or not. He has cured this kind of aneurism lately by firm pressure with a coin, old linen, and a tight roller. Dr. B. is, no doubt, a good surgeon, a good anatomist—but not very laconic.

5. *Medical Remuneration.* Sir A. Carlisle has put forth, in the TIMES newspaper, a proposed scale of medical fees, not quite so chimerical as some of his recent speculations. But it won't do. If practicable, it would be unjust—if just, it would be impracticable. The principle of the plan hinges on length of service, as in the army or navy—or rather on *seniority*.

*Physicians.*

"For every occasion of giving advice, or for visiting attendances, during the first five years of practice—fee, half a sovereign.

"For each of the same occasions after the first five years of practice, and until the end of twenty years from its commencement—one sovereign.

"After the experience of twenty years in continued practice, every physician is to be regarded as a referee, and when so employed in consultations, his fee to be two sovereigns, provided he does not assume the daily charge of the patient.

"Payments for journies to remain as they now stand.

"*Surgeons' Fees.*

"For each time of advice, attendance, or ministration, during the first five years—five shillings.

"After ten years of continued experience—half a sovereign.

"From the end of ten years—one sovereign.

"For operations dangerous to life, a scale should be fixed, tending to repress unjustifiable enterprises. I think the plan of daily fees would prove the best security.

"Payments for journies to remain as they now stand.

"*Apothecaries' Fees.*

"All apothecaries who practise as subphysicians, and hold the entire responsibility of treating patients, to be paid by fees, and the frequency of their visits to be at the discretion of their patients. It should be understood that those fees supersede charges for medicines.

"For each time of giving advice or attendance during the first five years of practice—half a crown.

"After five years and onward to ten years—fee five shillings.

"For ten years and onward—ten shillings."

REMARKS. There is one radical defect in this principle of remuneration, which vitiates it irrevocably. Sir A. seems to forget that man is, alas! doomed to *second* childhood; and therefore he has not taken into account the *decrement* of knowledge which takes place towards the close of life, as regularly as the *increment* in the earlier stages. Thus, suppose a physician commences practice at the age of 26 years, which is soon enough; his services, at the age of 45, (the prime of his faculties,) would be only worth half the remuneration of a man at the age of 70 or 75! This objection applies, *à fortiori*, to surgeons. Sir A. must add a descending scale.

In respect to the GENERAL PRACTITIONER, the difficulty of arranging his remuneration is almost insurmountable. It has occupied the thoughts of the writer of these remarks for 20 years past, and he cannot see any effectual remedy for the evils which now exist. The patient complains of too much medicine being ordered, when the remuneration depends on the medicines; but he would complain of there being too little medicine, if the latter were to be included in the fee, according to Sir A's scale. If, on the other hand, the general practitioner abandons entirely the dispensing of medicine, and charges merely for his visits, he hazards a fearful experiment, and multiplies tenfold the profit of the chemist, whose influence is too preponderant already! In the present state of things, it will be the wisest policy of the GENERAL PRACTITIONER to persevere in the attainment of the best possible EDUCATION; and when he enters on practice, to devote himself, with all his energy, to the cultivation of his profession, not only by careful observation, but by unremitted STUDY. We have no hesitation in saying that the great error of medical students is that of (comparatively speaking) abandoning study as soon as they have passed the "HALL." Yet that is the very period when real study should commence. Attendance on lectures, walking of hospitals, dissection of bodies, is not *study*. It is the mere imbibition of ideas from others—or rather it is learning the medical Alphabet. When the turmoil of lectures is over, then it is that the student should begin to digest the hurried meal which he has bolted down so expeditiously. Previously to this, he has not time to think, to read, to compare. If a young man is perceived to be *studious* when at home, and *observant* when visiting the sick, he will succeed in spite of all the bad laws and absurd system of medical remuneration, by which he is surrounded. But if he trusts to dress, address, whist-parties, and scandal-bearing, he may loudly declaim against the laws, whilst the fault is nearer home!

We confidently predict (and we do not speak from light authority) that the system of medical apprenticeship will be done away in an early part of the next session of parliament. This will be one of the most important measures for liberalising and improving the medical profession, that has ever been put in execution. This apprenticeship is not merely a waste of five years, but it is the foundation of an illiterate and unscientific disposition through life. It is almost impossible that the education of the general practitioner can be complete, or his station in society so good as it ought to be, while this mark of mechanical degradation is indented on parchment. No man should *bind* his son, for five years, behind a counter, who does not intend him to remain there as a chemist, all his life. As the Apothecaries' Company will, even now, admit five years' study, as an equivalent to five years' apprenticeship, (a great condescension certainly!) that man is mad, who would place his son under indenture, except on the express stipulation that he is to be free, after a year or two, to repair to the proper marts for medical science and literature.

6. *Strictura Orificii Urethræ.* Newton discovered the law of gravitation by the fall of an apple—Mr. Earle, the cause of a number of troublesome complaints, by being "disturbed in the act of making water." This cause is a natural contraction, either immediately at the orifice of the urethra, or a very little way from the orifice, behind which contraction, the urethra is of its natural diameter. The effects of this contraction are very deleterious, producing all the symptoms of the worst strictures, and *strictures themselves.* Irritable bladder, disease of the prostate, chronic affection of the testicles (sclerocele, or hydrocele) and great constitutional derangement, are the common consequences of this natural contraction of the orificium urethræ. These consequences are thought to be produced by the distention occasioned in the urethra from the check given to the current near the end of the passage. Mr. Earle thinks it induces far more serious disease in the urinary organs, and their appendages than stricture at any other part of the urethra. The remedy is simple—a division of the contraction, by means of an instrument which this ingenious surgeon has invented, and with which he operates in Bartholomew's Hospital.

---

### HOSPITAL PRACTICE.

32

### Foundling Hospital. M. Baron, Physician.

1. *Gastro-Enteritis of Infants.* Mr. Savatier reports from the above hospital on the subject of gastric and intestinal inflammation among newly born children. The following case is introduced from one of the wards of M. Baron, as an example of what the reporter denominates: "the first (slightest) degree of gastro-enteritis," in this class of subjects. On the 21st of April, was seen in the above ward, a child about six weeks old, presenting the following symptoms:—tension, and general, though moderate heat of the abdomen—intense redness of the anus and vicinity—tongue red at the sides and tip, white at the back and middle part—diarrhœa of greenish matters—constant, and almost tetanic rigidity of the muscles about the back of the neck—countenance expressive of suffering. *Abstinence—fomentations to the abdomen—syrup of mallows with gum.* In ten days, the symptoms were all dispersed. Each day the tongue was observed to be less red, the belly less tense, the rigidity of the muscles above-mentioned less tetanic.

In the winter of 1824-5, M. Savatier states, that he attended a great number of children affected with convulsions. It was observed, that these children first shewed symptoms of disinclination for the breast; were very fretful; had the tongue red at the sides; the belly tense and hot; the anus red, with moderate
